# A Mature Tertiary Lymphoid Structure with a Ki-67-Positive Proliferating Germinal Center Is Associated with a Good Prognosis and High Intratumoral Immune Cell Infiltration in Advanced Colorectal Cancer

**DOI:** 10.3390/cancers16152684

**Published:** 2024-07-28

**Authors:** Natsumi Mori, Gendensuren Dorjkhorloo, Takuya Shiraishi, Bilguun Erkhem-Ochir, Haruka Okami, Arisa Yamaguchi, Ikuma Shioi, Chika Komine, Mizuki Endo, Takaomi Seki, Nobuhiro Hosoi, Nobuhiro Nakazawa, Yuta Shibasaki, Takuhisa Okada, Katsuya Osone, Akihiko Sano, Makoto Sakai, Makoto Sohda, Takehiko Yokobori, Ken Shirabe, Hiroshi Saeki

**Affiliations:** 1 Department of General Surgical Science, Graduate School of Medicine, Gunma University, Maebashi 371-8511, Japan; m2000513@gunma-u.ac.jp (N.M.); m2220603@gunma-u.ac.jp (G.D.); m11201017@gunma-u.ac.jp (M.E.);; 2 Division of Gene Therapy Science, Gunma University Initiative for Advanced Research (GIAR), Maebashi 371-8511, Japan

**Keywords:** immune cell infiltration, locally advanced colorectal cancer, tertiary lymphoid structures

## Abstract

**Simple Summary:**

Tertiary lymphoid structures (TLS) arise in non-lymphoid tissues due to inflammation or cancer and play a key role in adaptive immune responses. In this study, we analyzed the TLS maturity in 78 patients with pathological T4 colorectal cancer (CRC). Mature TLS, identified by organized T (CD3+) and B (CD20+) lymphocytes with Ki-67-positive B cells, have been linked to microsatellite instability and improved cancer-specific and post-recurrence survival. High tumor Ki-67 expression correlated with poorer outcomes. The absence of mature TLS independently predicted poor survival. Tumors with mature TLS showed a higher infiltration of CD3+ T cells, FOXP3+ T cells, and CD86+ immune cells, including M1-like macrophages. Focusing on the Ki-67 expression pattern, the simultaneous evaluation of TLS maturity and tumor proliferation potency is suggested to be a potential prognostic indicator in CRC.

**Abstract:**

Tertiary lymphoid structures (TLSs) are complex lymphocyte clusters that arise in non-lymphoid tissues due to inflammation or cancer. A mature TLS with proliferating germinal centers is associated with a favorable prognosis in various cancers. However, the effect of TLS maturity on advanced colorectal cancer (CRC) remains unexplored. We analyzed the significance of TLS maturity and tumor Ki-67 expression in surgically resected tumors from 78 patients with pathological T4 CRC. Mature TLS was defined as the organized infiltration of T and B cells with Ki-67-positive proliferating germinal centers. We analyzed the relationship between TLS maturity and intratumoral immune cell infiltration. Mature TLS with germinal center Ki-67 expression was associated with microsatellite instability and improved survival; however, high tumor Ki-67 expression was associated with poor survival in the same cohort. Multivariate analysis identified the absence of mature TLS as an independent predictor of poor post-recurrence overall survival. Intratumoral infiltration of T lymphocytes and macrophages was significantly elevated in tumors with mature TLS compared to those lacking it. High Ki-67 levels and absent mature TLS were identified as poor prognostic factors in advanced CRC. Mature TLS could serve as a promising marker for patients at high-risk of CRC.

## 1. Introduction

Colorectal cancer (CRC) is one of the leading causes of cancer-related deaths globally [1]. However, the prognosis of advanced CRC with therapeutic resistance and complex heterogeneity is often poor despite advancements in surgical techniques and adjuvant chemotherapy [2,3]. Consequently, there is an urgent need to identify reliable biomarkers to predict patient outcomes and guide therapeutic strategies more effectively. This approach is expected to resolve important issues in the improvement of clinical CRC care.

Recently, many researchers have highlighted the significance of the tumor microenvironment (TME) in cancer progression, patient prognosis, and therapeutic resistance [4]. Among the various components of the TME, tertiary lymphoid structures (TLSs) have gained attention for their potential role in predicting patient prognosis and enhancing antitumor immunity through antigen presentation and production of tumor-specific antibodies [5,6]. These structures, which resemble secondary lymphoid organs, including the spleen and lymph nodes, are composed of B and T cell zones and are found in chronically inflamed or cancerous tissues [7]. It has been reported that the presence of TLS in tumor tissues is associated with good prognosis in several cancers, including CRC [8,9,10,11]. In contrast, not only TLS densities but also maturity levels have been reported to be important in the regulation of antitumor immunity, which contributes to the prognosis and treatment resistance of cancer patients [12,13].

Mature TLSs are characterized by the presence of well-developed germinal centers with proliferating B cells [10,14]. These structures have been reported to facilitate robust antigen presentation and antibody production, thereby enhancing immunotherapy sensitivity and antitumor immunity within the TME. In cancer research, high expression levels of the proliferation marker Ki-67 in cancer cells are well known to be related to cancer aggressiveness and poor prognosis in various cancers [15,16,17,18,19]. Therefore, the evaluation of Ki-67 within the TME suggests distinguishing not only TLS maturity but also cancer cell aggressiveness, providing a possibility for a clearer understanding of the immune landscape and cancer characteristics simultaneously. However, the prognostic value of the simultaneous evaluation of tumor Ki-67 expression and mature TLS with germinal center Ki-67 expression in patients with advanced CRC remains unclear. 

This study aimed to clarify the relationship between TLS maturity, tumor Ki-67 expression, clinicopathological factors, and tumor-infiltrating T lymphocytes, macrophages, and B lymphocytes in patients with advanced CRC. Therefore, we performed histochemical staining for the T lymphocyte marker CD3, B lymphocyte marker CD20, and proliferation marker Ki-67 to identify the maturity of peritumoral TLS in surgically resected specimens from patients with pathological T4 (pT4) CRC. 

## 2. Materials and Methods

### 2.1. Clinical Samples 

This study enrolled 78 patients diagnosed with pT4 CRC who underwent curative resection at Gunma University Hospital between July 2013 and February 2020. Of the 78 patients, 50 received adjuvant chemotherapy after surgery. The exclusion criteria were preoperative treatment and non-curative resection due to distant metastasis. Relevant clinical data were retrieved from the medical and surgical records.

### 2.2. Immunohistochemical Staining

Paraffin-embedded CRC specimens were cut into 4 µm thick sections. These sections were incubated at 60 °C for 60 min and deparaffinized using ClearPlus (FALMA, Tokyo, Japan). Rehydration was carried out using a series of ethanol treatments and antigen retrieval using Immunosaver (Nishin EM, Tokyo, Japan) at 98–100 °C for 45 min. Sections were treated with 0.3% hydrogen peroxide in 100% methanol for 30 min at 20–25 °C to inhibit endogenous peroxidase activity. Subsequently, the sections were blocked with Protein Block Serum-Free Reagent (Agilent, Santa Clara, CA, USA) and exposed to primary antibodies in REAL Antibody Diluent (Agilent, Santa Clara, CA, USA) at 4 °C for 24 h. The following primary antibodies were used: CD3 (1:1; Ventana, Tucson, AZ, USA; 790-4341), CD8 (1:400; Abcam, Cambridge, UK; ab4055), FOXP3 (1:500; Abcam, Cambridge, UK; ab20034), CD163 (1:500; Cell Signaling Technology, Danvers, MA, USA; CST-93498S), CD86 (1:400; Cell Signaling Technology, Danvers, MA, USA; CST-91882S), Ki-67 (1:500; Cell Signaling Technology, Danvers, MA, USA; CST-9027S), and CD20 (1:1; Ventana, Tucson, AZ, USA; 760-2531). The primary antibody was visualized using a Histofine Simple Stain MAX-PO (Multi) Kit (Nichirei, Tokyo, Japan), following the manufacturer’s instructions. The chromogen 3,3-diaminobenzidine tetrahydrochloride was applied at a concentration of 0.02% in 50 mM ammonium acetate-citrate buffer (pH 6.0) containing 0.005% hydrogen peroxide. Finally, Mayer’s hematoxylin was used for counterstaining. Negative controls were incubated without the primary antibody and showed no detectable staining.

### 2.3. Evaluation of Peritumoral Mature TLS 

**Figure 1:**
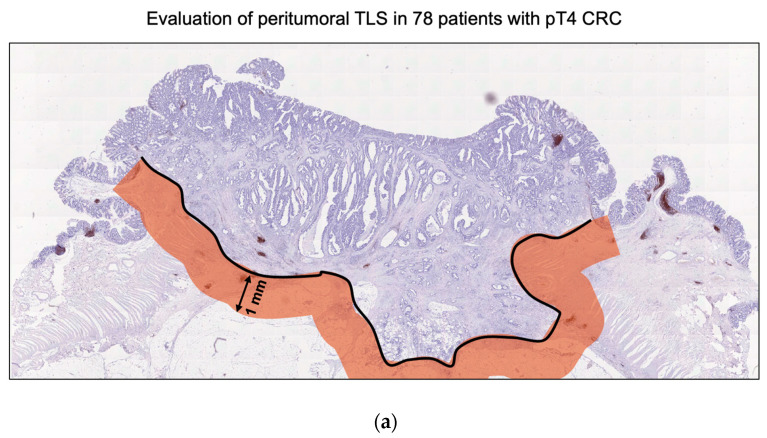

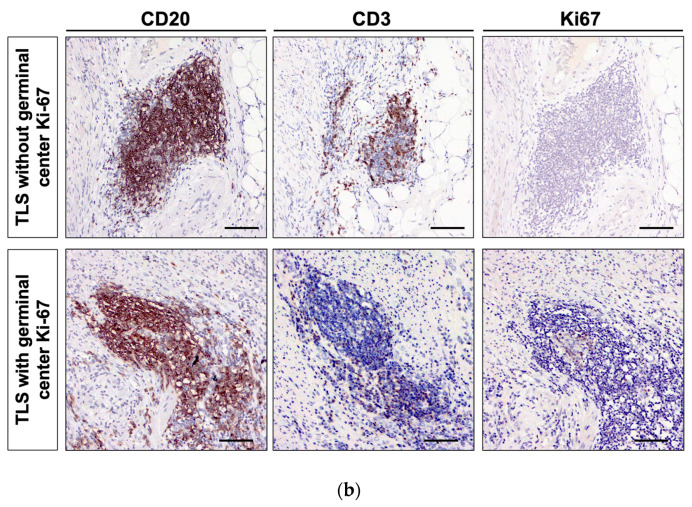
Evaluation of mature TLS with germinal center Ki-67 in pathological T4 (pT4) colorectal cancer (CRC) samples. (**a**) The area 1 mm from the invasion front of the pT4 CRC was defined as the peritumoral area. This study evaluated the significance of TLS in the peritumoral area. (**b**) Representative images of pan-T cell marker CD3, B cell marker CD20, and proliferation marker Ki-67 in pT4 CRC specimens. Upper panel: TLS without Ki-67-positive proliferating B cells in the germinal center was defined as immature TLS. Lower panel: TLS with Ki-67-positive proliferating B cells in the germinal center was defined as mature TLS. Images were captured at 200× magnification. Scale bar, 100 µm.

Sequential sections of surgical specimens from pT4 CRC were immunohistochemically stained for CD20, CD3, and Ki-67 to assess the presence and maturity of TLS in the TME. TLSs were identified by clustering both B cells (CD20+) and T cells (CD3+). The maturity of TLS was determined by evaluating nuclear Ki-67 expression in immune cells within the germinal center. TLS containing germinal centers with nuclear Ki-67-expressing immune cells were classified as mature cells. If one Ki-67-positive TLS was found in the peritumoral area, especially within 1 mm of the invasive margin, it was considered positive (Figure 1). 

### 2.4. Evaluation of Tumoral Ki-67 Expression in Tumor Tissues

Tumoral Ki-67 expression was assessed in sections of surgical specimens that were immunohistochemically stained for Ki-67. Images of five representative fields were captured at 200× magnification using a microscope (BZ-X700; Keyence, Osaka, Japan). The Ki-67 expression in these images was manually quantified using a Java-based image processing software (ImageJ 1.53; National Institutes of Health, Bethesda, MD, USA). We specifically evaluated the nuclear Ki-67 expression in 100 cancer cells per image, totaling 500 cells per sample. The average value across the five fields was computed for each patient. Furthermore, Ki-67 expression levels were classified into two categories based on the ROC curve for disease-free survival, with a cut-off value of 22.8 (Ki-67 low, n = 68; Ki-67 high, n = 10).

### 2.5. Image Acquisition and Quantitative Evaluation of Immune Cells 

To count the tumor-infiltrating immune cells (CD3+, CD8+, FOXP3+, CD86+, and CD163+), we captured four fields from the tumor section, encompassing 36 images covering 9.070624 mm^2^, using a microscope (BZ-X700; Keyence, Osaka, Japan). A Hybrid Cell Count System (Keyence, Osaka, Japan), a semi-automatic image analysis software, was used to count immune cells in digital images. The density of tumor-infiltrating immune cells was calculated by dividing the number of cells by the total area (mm^2^), yielding cell density per mm^2^.

### 2.6. Statistical Analysis 

Chi-squared and Fisher’s exact tests were used to examine the relationships between categorical values. The Mann–Whitney U test was used to compare the means of continuous variables across different groups. Survival curves were visualized using Kaplan–Meier curves with the log-rank test to assess differences between groups. Cox regression analyses, both univariate and multivariate, were conducted to identify the independent predictors of post-recurrence overall survival. Statistical analyses were performed using JMP Pro 15 (SAS Institute, Cary, NC, USA) and GraphPad Prism 10 (Dot Matics, Boston, MA, USA). Statistical significance was defined as *p* < 0.05.

## 3. Results

### 3.1. Evaluation of Distribution and Maturity of TLS in pT4 CRC Samples 

To clarify the significance of TLS maturity in pT4 advanced CRC, we defined a 1 mm area from the invasion front line of the tumor tissue as the peritumoral area. Next, we immunohistochemically evaluated the distribution of TLS in this area as previously described (37016103) (Figure 1a). The histological characteristics of TLS were identified as a lymphoid tissue structure with T cells surrounding the germinal center B cells, using the T cell marker CD3 and B cell marker CD20. This study defined TLS with germinal center Ki-67 in the peritumoral area as mature TLS and TLS without Ki-67 as immature TLS (Figure 1b). Among the 78 pT4 CRC samples, 76 (97.4%, 76/78) exhibited the presence of at least one peritumoral TLS, with an average of 8.7 (±6.6) TLSs observed in each specimen. Additionally, 30 (38.5%, 30/78) demonstrated the coexistence of mature and immature TLSs. Based on the presence of mature TLS, 30 (38.5%, 30/78) were classified into the mature TLS group and 48 (61.5%, 48/78) into the immature TLS group (Table 1).

### 3.2. Association of Mature TLS with the Clinicopathological Features and Survival of Clinical Advanced CRC Patients

Table 1 shows the relationship between mature TLS, germinal center Ki-67, and patient clinicopathological characteristics. The positivity of mature TLS was significantly associated with microsatellite status, which has been reported to play a significant role in activating antitumor immunity (*p* = 0.0081) (Table 1).

**Figure 2:**
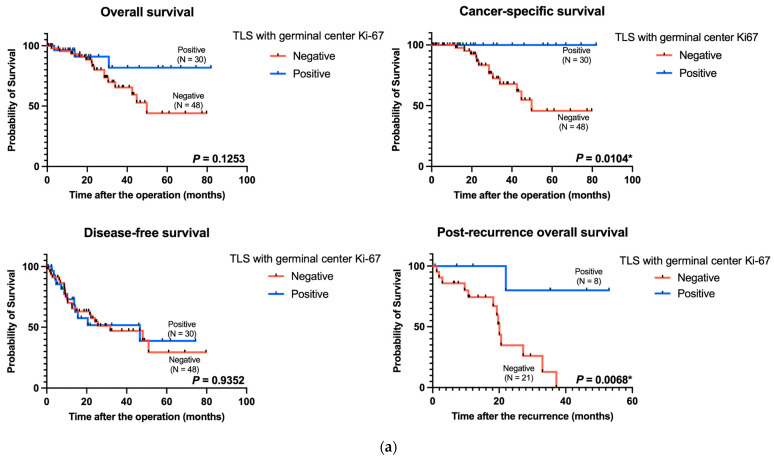

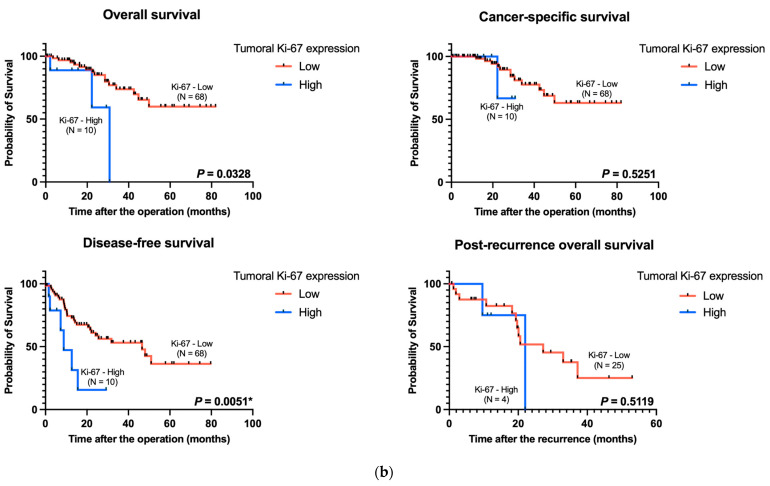
Kaplan–Meier curves of the data from patients with pT4 CRC based on TLS maturity and tumoral Ki-67 expression. (**a**) Kaplan–Meier survival analyses for overall survival (*p* = 0.1253, left upper panel), cancer-specific survival (*p* = 0.0104, right upper panel), disease-free survival (*p* = 0.9352, left lower panel), and post-recurrence overall survival (*p* = 0.0068, right lower panel) stratified according to TLS maturity. (**b**) Kaplan–Meier survival analyses for overall survival (*p* = 0.0328, left upper panel), cancer-specific survival (*p* = 0.5251, right upper panel), disease-free survival (*p* = 0.0051, left lower panel), and post-recurrence overall survival (*p* = 0.5119, right lower panel) stratified according to tumoral Ki-67 expression. TLS: tertiary lymphoid structure. * *p*-value < 0.05.

We explored the prognostic impact of mature TLS with germinal center Ki-67 expression using survival analyses of overall survival, cancer-specific survival, and disease-free survival in 78 patients with surgically resected pT4 CRC. CRC and mature TLS were significantly associated with prolonged cancer-specific survival (*p* = 0.0104) (Figure 2a). Moreover, patients with recurrent CRC (n = 29) with mature TLS had better post-recurrence overall survival than did those without mature TLS (*p* = 0.0068) (Figure 2a). The differences were not significant in terms of disease-free and overall survival (Figure 2a).

The high expression levels of tumoral Ki-67 were related to poor overall survival and disease-free survival in our cohort (*p* = 0.0328 and *p* = 0.0051, respectively) (Figure 2b). However, the difference was not significant in terms of cancer-specific survival and post-recurrence overall survival (Figure 2b). 

Table 2 shows the results of the multivariate analysis of post-recurrence overall survival using the Cox regression model. Multivariate analysis revealed that negativity for mature TLS was an independent predictor of shorter post-recurrence overall survival (HR = 32.546, 95% CI: 2.8759–368.31, *p* = 0.0049) (Table 2). 

### 3.3. Correlation between Mature TLS with Germinal Center Ki-67 and Immune Cell Infiltration in Advanced CRC Tumors

**Figure 3:**
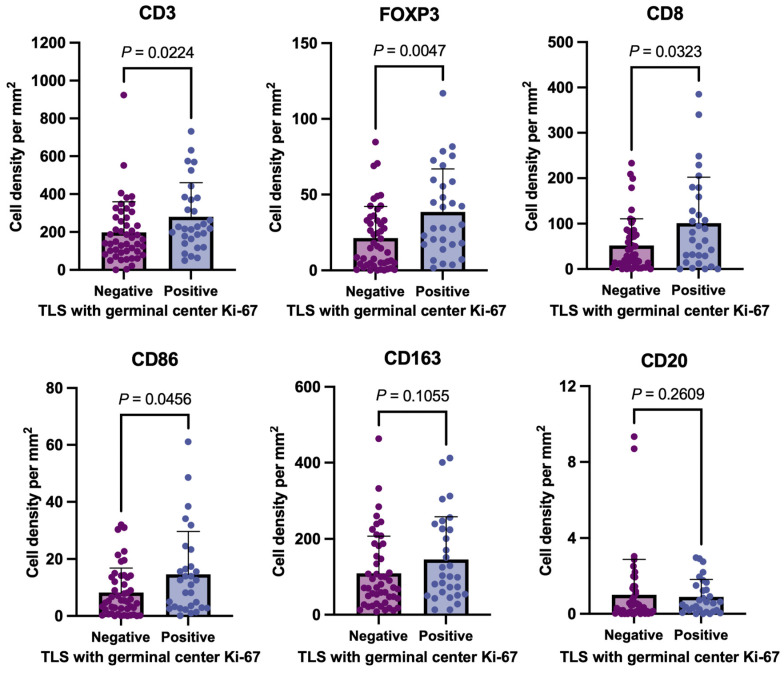
Relationship between immune cell infiltration and TLS maturity in 78 advanced pT4 CRC tissues. This figure compares the intratumoral infiltration levels of CD3+ (T-cell marker), FOXP3 (regulatory T-cell marker), CD8+ (cytotoxic T-cell marker), CD86+ (immune cells including M1-like macrophages), CD163+ (anti-inflammatory macrophage marker), and CD20+ (B-cell marker) immune cells between the negative and positive groups of mature TLS with Ki-67-positive proliferating germinal centers. TLS: tertiary lymphoid structure.

Figure 3 presents the relationships between mature TLS and germinal center Ki-67 and intratumoral CD3+, FOXP3+, CD8+, CD86+, CD163+, and CD20+ immune cells. Tumors with mature TLS were associated with more intratumoral CD3+, FOXP3+, and CD8+ lymphocytes and CD86+ immune cells including M1-like macrophages than those without mature TLS (Figure 3). The tumors with mature TLS were associated with more intratumoral FOXP3+ lymphocytes and CD86+ macrophages among MSS tumors (n = 66, Supplementary Figure S1A) and MSI tumors (n = 12, Supplementary Figure S1B), respectively.

## 4. Discussion

This study clarified that the presence of peritumoral mature TLS was associated with microsatellite instability and improved cancer-specific and post-recurrence overall survival; however, high expression of tumoral Ki-67 was related to poorer disease-free and overall survival in the same cohort. Moreover, multivariate analysis identified the negativity of peritumoral mature TLS as an independent predictor of poor post-recurrence overall survival in patients with advanced pT4 CRC. In addition, intratumoral infiltration levels of T lymphocytes, FOXP3+ regulatory T cells, and CD86+ immune cells including M1-like macrophages were significantly higher in tumors with mature peritumoral TLS than in those without mature TLS.

In this study, patients with pT4 CRC with mature TLS had better cancer-specific and overall survival rates than did those without mature TLS, although there was no significant difference in disease-free survival. Moreover, we showed that patients with CRC and mature TLS had better post-recurrence survival compared to patients with CRC but without mature TLS. Therefore, it has been proposed that local tumor immune activation by mature TLS contributes minimally to preventing recurrence in locally advanced CRC. However, TLS status is associated with the response to treatment following recurrence. Evaluating TLS maturity may be useful for predicting the therapeutic response in patients with advanced CRC who experience recurrence.

In various gastrointestinal cancers, higher density and maturity of TLS in tumors have been linked to favorable outcomes [20,21,22]. Previously, researchers have evaluated TLS density and maturation within the tumor and peritumoral regions. However, among patients with CRC without metastatic regions, peritumoral TLS, rather than intratumoral TLS, demonstrated a strong predictive value for patient prognosis [8]. They also showed that intratumoral TLSs were detected in a minority of cases, and their presence or density did not correlate with patient prognosis. Similarly, Ding et al. showed that peritumoral TLSs exhibit favorable prognostic implications in intrahepatic cholangiocarcinoma [23]. Thus, this study focused on peritumoral TLS to analyze the relationship between TLS maturity and clinicopathological significance in our CRC cohort. Furthermore, the originality and novelty of this study are that we focused on the relationship between peritumoral TLS maturity and intratumoral immune cell infiltration in a unique cohort of pT4 locally advanced CRC specimens rather than in typical Stage I-III CRC specimens. 

Ki-67 is a widely recognized marker used to assess the proliferation rate of tumor cells [24]. In CRC, elevated Ki-67 expression is associated with increased tumor aggressiveness and poor prognosis [25,26]. Similar to previous findings, higher Ki-67 expression was associated with significantly shorter disease-free survival and overall survival in patients with pT4 CRC. In addition to providing information on tumor proliferation and aggressiveness, Ki-67 can be used to assess the local immune response against cancer. Based on these findings, Ki-67 evaluation is a simple and informative method that can be used as a biomarker for assessing cancer aggressiveness and TLS maturity in CRC. 

Mature TLSs play a crucial role in influencing immune cell infiltration into the TME [9]. An integral part of the TLS regulates the trafficking and recruitment of T cells, B cells, and macrophages through several mechanisms, such as the secretion of peripheral node addressin, mucosal addressin cell adhesion molecule-1 (MAdCAM-1), and L-selectin ligands [27,28,29]. For instance, MAdCAM-1 acts as a potent recruiter of immune cells through its interaction with the α4β7 integrin present in immune cells, including T cells and macrophages [30,31,32]. In this study, mature TLSs with Ki-67 expression were associated with a significantly higher intratumoral infiltration of CD3-, CD8-, FOXP3-, and CD86-positive cells. These findings suggest that mature TLS may have a significant impact on peritumoral immune cell infiltration through several mechanisms, including the formation of venules and the secretion of adhesion molecules, selectins, and addressins. Interestingly, although we found a significant correlation between the presence of peritumoral mature TLS and a higher number of CD86+ cells (including M1-like macrophages) in tumors, there was no significant difference in CD163+ cells (a marker for M2-like macrophages). Cytokines such as interferon (IFN)-γ and tumor necrosis factor (TNF)-α, secreted by abundant intra-tumoral T cells within mature TLS, promote a pro-inflammatory M1 phenotype polarization [33,34]. These findings indicate that mature TLS might regulate the M1-like polarization and infiltration of intra-tumoral macrophages via cytokines such as IFN-γ and TNF-α secreted by T cells and addression/L-selectin. Taken together, these results suggest that the TLS may not only play a role in immune cell recruitment but also be crucial for delicately balancing the TME. 

This study has certain limitations. First, it was conducted retrospectively at a single institution and focused on patients with surgically resected pT4 CRC. Moreover, the sample size of the cohort was limited to 78 patients. This relatively small cohort may have introduced sampling bias. Our findings may not fully capture the importance of mature TLS in all patients with CRC, including those with inoperable disease. Second, we did not evaluate the regulators related to TLS formation, such as CXCL13 and MAdCAM-1, which are primarily involved in the mechanisms that attract immune cells to the TLS. Investigating the levels and dynamics of these regulators may provide valuable insights into the formation and regulation of TLS in CRC. Future research should consider comprehensive analyses to elucidate the biology of TLS in CRC. Moreover, additional research, including patients with recurrent/unresectable CRC, is required to assess whether detecting mature TLS in pretreatment biopsy samples could predict the response to chemotherapy.

## 5. Conclusions

Our findings have potential implications for future CRC treatments. By focusing on the Ki-67 expression pattern, we identified two poor prognostic factors in advanced CRCs: high tumor Ki-67 expression and a lack of mature TLS with germinal center Ki-67 expression. The presence of mature TLS is associated with high infiltration of intratumoral immune cells and better survival in patients with pT4 CRC. This suggests that TLS maturity is an important regulator of intratumoral immune cell infiltration, and the evaluation of mature TLS could serve as a promising predictor for identifying high-risk CRC patients. 

## Figures and Tables

**Table 1:** The relationship of clinicopathological factors and TLS with germinal center Ki-67 in 78 patients with pT4 CRC.

Variable	TLS with Germinal Center Ki-67		*p-*Value
	Negative	Positive	
	N = 48	N = 30	
Age (y)			
<65	15 (31.2)	10 (33.3)	0.8481
≥65	33 (68.8)	20 (66.7)	
Sex			
Female	23 (47.9)	12 (40.0)	0.4932
Male	25 (52.1)	18 (60.0)	
Microsatellite status			
MSS	45 (93.8)	21 (70.0)	0.0081 *
MSI	3 (6.2)	9 (30.0)	
Tumor location			
Colon	32 (66.7)	22 (73.3)	0.5326
Rectum	16 (33.3)	8 (26.7)	
Pathological T stage			
pT4a	27 (56.3)	14 (46.7)	0.4095
pT4b	21 (43.7)	16 (53.3)	
Tumor size (mm)			
Small (<50)	19 (39.6)	12 (40.0)	0.9708
Large (≥50)	29 (60.4)	18 (60.0)	
Desmoplastic reaction			
Mature	17 (35.4)	12 (40.0)	0.6841
Intermediate or immature	31 (64.6)	18 (60.0)	
Tumoral Ki-67 expression			
Low	43 (89.6)	25 (83.3)	0.4272
High	5 (10.4)	5 (16.7)	
Lymph node metastasis			
Absence	11 (22.9)	11 (36.7)	0.1925
Presence	37 (77.1)	19 (63.3)	
Distant metastasis			
Absence	38 (79.2)	26 (86.7)	0.3934
Presence	10 (20.8)	4 (13.3)	
Radical resection margin			
Negative	40 (83.3)	25 (83.3)	1.0000
Positive	8 (16.7)	5 (16.7)	
Adjuvant chemotherapy			
Presence	30 (62.5)	20 (66.7)	0.7084
Absence	18 (37.5)	10 (33.3)	

TLS: tertiary lymphoid structure, MSI: microsatellite instability, MSS: microsatellite stable, * *p*-value < 0.05.

**Table 2:** Multivariate analysis for post-recurrence overall survival in pT4 CRC patients with recurrence.

Factors	Univariate Analysis			Multivariate Analysis		
	HR	95% CI	*p-*Value	HR	95% CI	*p-*Value
Pathological T stage						
pT4a	1		0.3742	1		0.6892
pT4b	1.6447	0.5489–4.9279		1.3078	0.3510–4.8725	
Tumor size (mm)						
Small (<50)	1		0.5987	1		0.2382
Large (≥50)	0.7446	0.2482–2.2333		0.3706	0.0712–1.9288	
Microsatellite status						
MSS	1			1		0.1954
MSI	0.3459	0.0443–2.7008	0.3113	0.1575	0.0096–2.5857	
Lymph node metastasis						
Presence	1		0.4830	1		0.0730
Absence	0.5809	0.1273–2.6491		14.017	0.7820–251.27	
Distant metastasis						
Presence	1		0.5142	1		0.1589
Absence	0.7021	0.2426–2.0316		3.2386	0.6314–16.612	
Adjuvant chemotherapy						
Presence	1		0.3176	1		0.3840
Absence	1.7549	0.5824–5.2874		1.7698	0.4895–6.3983	
Tumoral Ki-67 expression						
Low	1		0.5164	1		0.7348
High	1.6696	0.3549–7.8534		1.563	0.1180–20.710	
TLS with germinal center Ki-67						
Positive	1		0.0256 *	1		0.0076 *
Negative	10.483	1.3325–82.468		26.286	2.3852–289.69	

TLS: tertiary lymphoid structure, MSS: microsatellite stable, MSI: microsatellite instability, HR: hazard ratio, CI: confidence interval, * *p*-value < 0.05.

## Data Availability

Derived data supporting the findings of this study are available from the corresponding author T.Y. on request.

## Supplementary Materials

The following supporting information can be downloaded at: https://www.mdpi.com/article/10.3390/cancers16152684/s1, Supplementary Figure S1: Relationship between immune cell infiltration and TLS maturity in MSS and MSI pT4 colorectal tumor tissues.
