# Supplementary material for: A Mature Tertiary Lymphoid Structure with a Ki-67-Positive Proliferating Germinal Center Is Associated with a Good Prognosis and High Intratumoral Immune Cell Infiltration in Advanced Colorectal Cancer
Source: Cancers (Basel). 2024 Jul 28;16(15):2684. doi: 10.3390/cancers16152684 (PMC11312168; doi:10.3390/cancers16152684)
Supplement: Supplementary file 1 [file cancers-16-02684-s001.zip › cancers-3111469-supplementary.pdf]

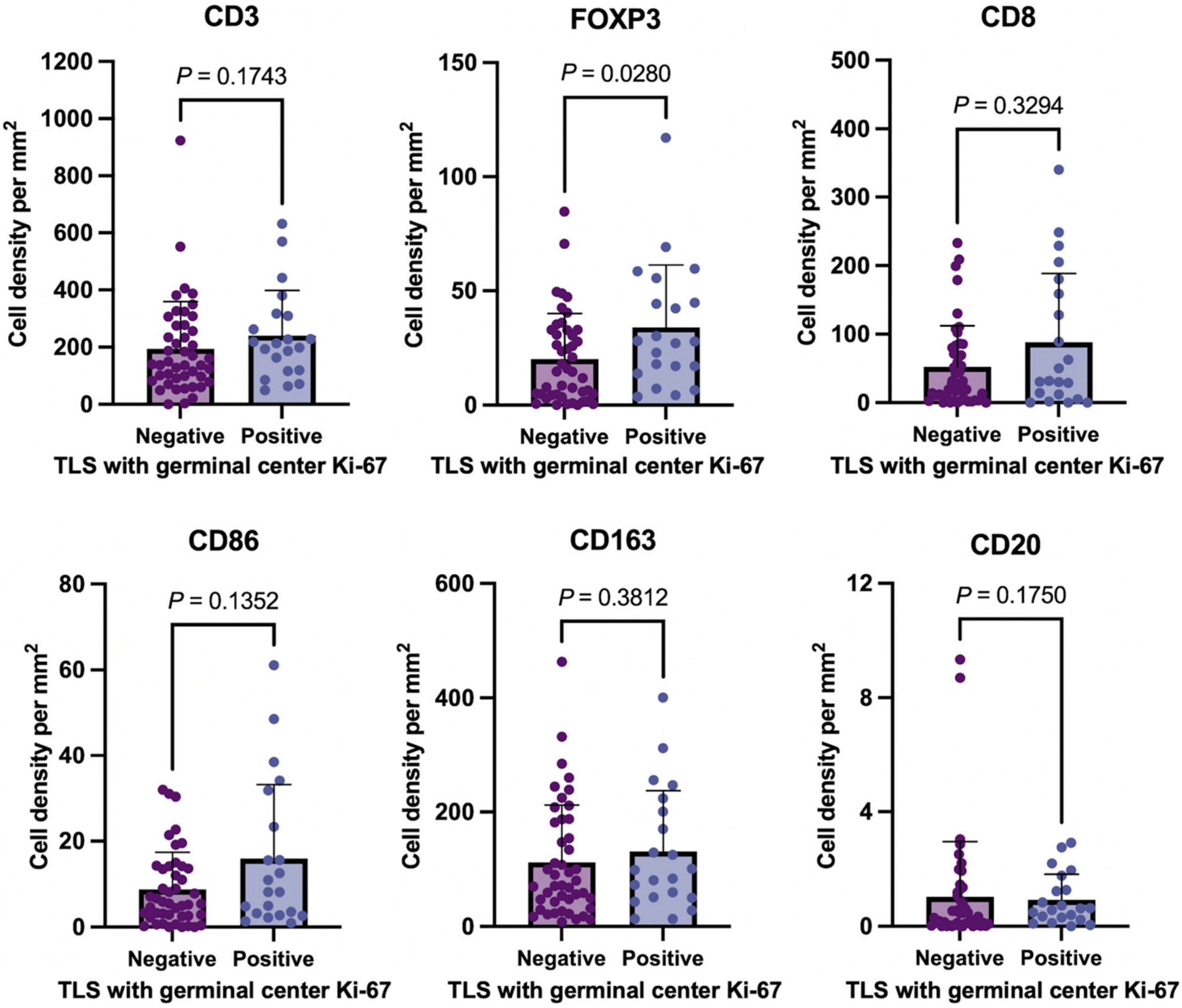
CD3
P = 0.1743
Cell density per mm2
1200
1000
800
600
400
200
0
Negative
Positive
TLS with germinal center Ki-67
FOXP3
P = 0.0280
Cell density per mm2
150
100
50
0
Negative
Positive
TLS with germinal center Ki-67
CD8
P = 0.3294
Cell density per mm2
500
400
300
200
100
0
Negative
Positive
TLS with germinal center Ki-67
CD86
P = 0.1352
Cell density per mm2
80
60
40
20
0
Negative
Positive
TLS with germinal center Ki-67
CD163
P = 0.3812
Cell density per mm2
600
400
200
0
Negative
Positive
TLS with germinal center Ki-67
CD20
P = 0.1750
Cell density per mm2
12
8
4
0
Negative
Positive
TLS with germinal center Ki-67

A

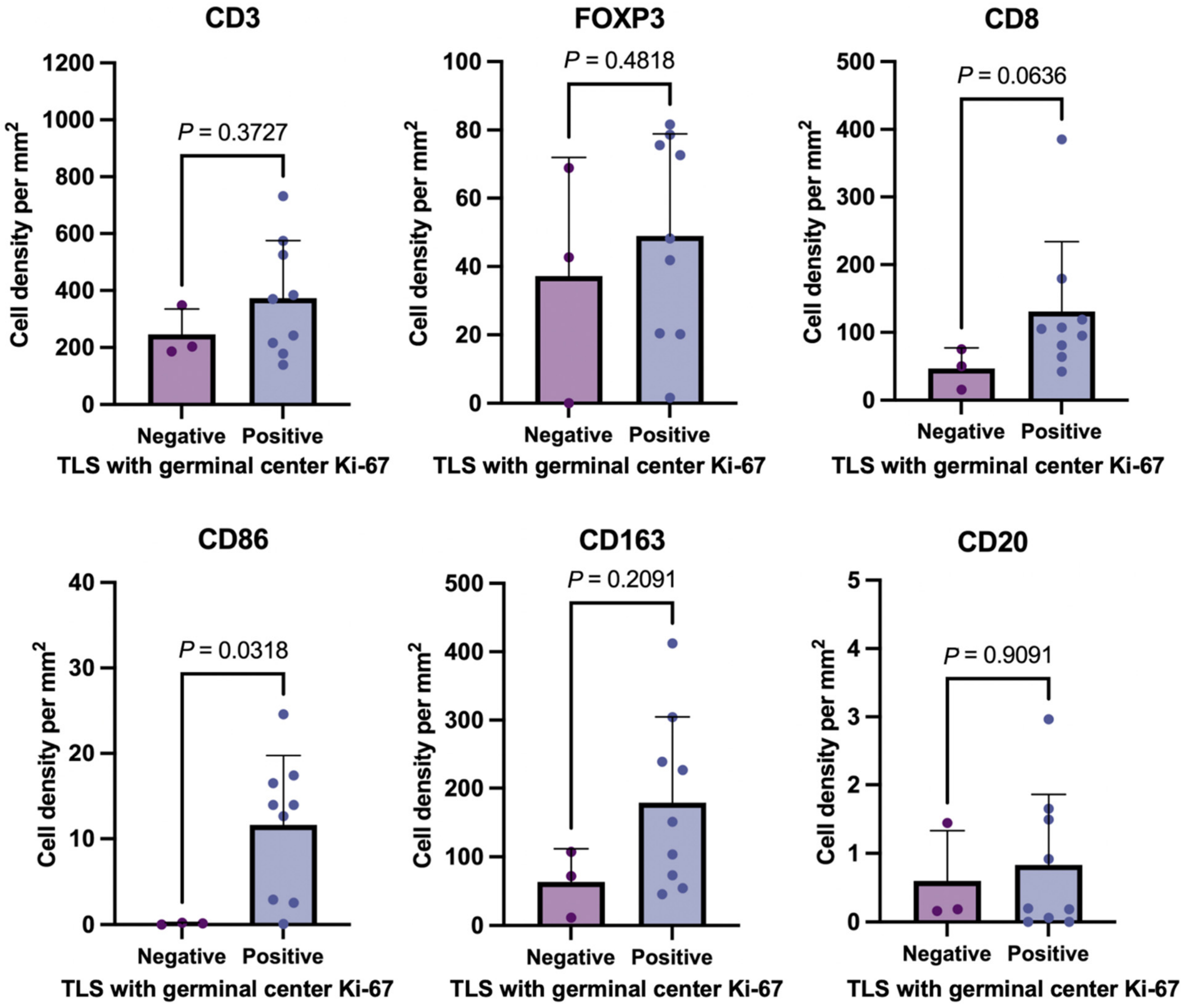

B

Supplementary Figure S1. Relationship between immune cell infiltration and TLS maturity in MSS and MSI pT4 colorectal tumor tissues. The panel (A) compares the intratumoral infiltration levels of CD3+ (T-cell marker), FOXP3 (regulatory T-cell marker), CD8+ (cytotoxic T-cell marker), CD86+ (including pro-inflammatory M1-like macrophage marker), CD163+ (anti-inflammatory macrophage marker), and CD20+ (B-cell marker) immune cells between the negative and positive groups of mature TLS with Ki-67 positive proliferating germinal centers in 66 patients with MSS tumors. The panel (B) compares the intratumoral infiltration levels of CD3+, FOXP3, CD8+, CD86+, CD163+, and CD20+ immune cells between the negative and positive groups of mature TLS with Ki-67-positive proliferating germinal centers in 12 patients with MSI tumors. TLS: tertiary lymphoid structure, MSS: microsatellite stable, MSI: microsatellite instability.
